# Leadership as decision architecture: an organizational framework for understanding and eliminating physical restraint in human services

**DOI:** 10.3389/fpsyg.2026.1889369

**Published:** 2026-07-22

**Authors:** Nicole Sorensen

**Affiliations:** 1 MyPath, Oconomowoc, WI, United States; 2 College of Health, Education & Social Welfare, University of Wisconsin-Green Bay, Green Bay, WI, United States

**Keywords:** decision ecology, implementation climate, intellectual disability, organizational leadership, physical restraint, positive behavior support, psychological safety, restraint reduction

## Abstract

Physical restraint persists at high rates in human services settings supporting individuals with intellectual and developmental disabilities (IDD), even within organizations that have formally adopted positive behavior support (PBS) frameworks and invested substantially in staff training. This perspective article introduces the Staff Decision Ecology Model, a decision ecology account of physical restraint, which conceptualizes restraint as a context-dependent, staff-mediated decision shaped by nested individual, environmental, and organizational variables, and argues that leadership functions as decision architecture: the organizational force that structures upstream conditions before any crisis arrives. Three empirically grounded mechanisms are examined: psychological safety, implementation climate, and team communication structures. A conceptual model is proposed, and a solutions-oriented agenda is advanced specifying leadership development targets, organizational assessment strategies, and a testable multilevel research program. The central argument is that restraint elimination requires organizational conditions that leaders build or fail to build, and that continued reliance on training-centered approaches, absent that organizational investment, will produce the same unsatisfying results.

## Introduction

The persistence of physical restraint in human services is among the most examined and least solved problems in the field. Decades of regulatory pressure, framework adoption, and training investment have produced meaningful progress in some contexts and almost none in others, often within the same organization. That variability is the field’s most important empirical signal and has not been sufficiently theorized.

The dominant explanatory frame remains individual and skill-based: restraint persists because staff lack fluency in de-escalation or have not received sufficient crisis prevention training. The frame is not wrong (skill matters), but it cannot account for why two units with comparable staff, comparable training, and comparable populations produce dramatically different restraint rates year over year.

Björne et al. (2023), in a survey of Swedish front-line managers in intellectual disability services, found that managers consistently reported lower restraint rates than the staff working under them, a perceptual gap the authors interpreted as evidence of organizational drift: the gradual normalization of restrictive practices within systems that nominally oppose them. Deveau et al. (2020), using Delphi methodology with senior managers in UK intellectual disability organizations, found that senior leadership focus remained almost entirely unstudied empirically despite being described as central to organizational functioning. Both findings point to the same conclusion: the organizational tier of the problem is real, proximate, and undertheorized.

This paper offers a framework for that theorization: the Staff Decision Ecology Model, which reconceptualizes restraint as a staff-mediated decision event shaped by the interacting tiers of antecedent context, staff internal processes, and organizational contingencies, together with a post-event feedback loop that drives either learning or drift. Within that model, this paper specifies leadership as the primary architect of the organizational tier. Restraint is in this sense an organizational behavior: organizational conditions, built or eroded by leadership, determine what frontline staff perceive as possible, sanctioned, and safe in a moment of behavioral crisis. The staff decision itself remains the proximal mechanism through which those conditions produce a restraint or a non-restraint outcome.

## The empirical case against training-only approaches

Training in crisis prevention and PBS produces measurable effects under controlled conditions. Mahon et al. (2021) found that multicomponent training packages incorporating modeling, role-play, and behavioral feedback produced the most robust skill acquisition across residential and day support settings. The problem is not that training fails in the training room; it is what happens when trained staff return to organizational contexts that do not reinforce or model the practices they have been taught. Hayward et al. (2021) identified organizational readiness, supervisory support, and leadership engagement as the primary moderators of whether training translated into sustained implementation, not training content itself.

Haines-Delmont et al. (2022) provide among the most direct empirical tests of an organizational approach. Implementing the No Force First framework across a large UK mental health organization serving individuals with learning disabilities, they documented a significant 17% reduction in physical restraint incidence (IRR = 0.83, 95% CI 0.77–0.88, *p* < 0.0001). No Force First is not a training curriculum; it is an organizational philosophy with explicit leadership requirements, structured governance, and non-punitive incident review. The training element is embedded within an organizational change model, not the other way around.

At the individual decision level, Ma et al. (2023) modeled the predictors of physical restraint intention among critical care nurses using structural equation modeling. Attitude (*β* = 0.29, *p* < 0.05), subjective norm (*β* = 0.25, *p* < 0.05), and perceived behavioral control (*β* = 0.32, *p* < 0.001) each independently predicted restraint intention. The subjective norm finding is particularly consequential: subjective norm reflects what staff believe their organizational community expects them to do in a crisis. That belief is not a training variable; it is built or eroded by leadership. Nunno et al. (2022), analyzing 26 years of restraint fatality data among children and adolescents in the United States, similarly identified organizational structures and processes, not individual skill deficits, as the primary explanatory variables.

## The Staff Decision Ecology Model

The Staff Decision Ecology Model conceptualizes physical restraint as a context-dependent behavioral decision, drawing on applied behavior analysis, implementation science, and the SEIPS 2.0 sociotechnical systems framework (Holden et al., 2013). It proposes that the decision to use restraint is the outcome of a work system in which the antecedent context, staff internal processes, and organizational contingencies interact dynamically, with a post-event feedback loop shaping subsequent decisions. No single variable is determinative; the ecology of interactions is.

Within staff internal processes, staff beliefs about challenging behavior, perceived self-efficacy, and prior experience with crisis shape how behavioral events are interpreted and what responses are generated. Within the antecedent context, staffing ratios, physical space, and access to backup support constrain options physically available in a crisis moment. These are not fixed conditions; they are products of organizational resource allocation and scheduling decisions. Within organizational contingencies, policy clarity, supervision quality, performance feedback patterns, and the relational climate encode, in the staff member who encounters a crisis, beliefs about what is available, what is sanctioned, and what is safe. This is the mechanism by which organizational conditions shape individual decisions without being proximate to them. Ntinas (2024) documented that staff burnout (itself an organizational outcome) significantly predicted resistance to practice change, illustrating the feedback loop in which organizational conditions erode the individual variables that restraint-free practice requires.

The model thus offers a different unit of analysis than training-centered approaches. Where training addresses individual skill acquisition, the decision ecology focuses on the organizational conditions that determine whether those skills are deployed in practice. Leadership is the primary determinant of that ecology.

The three organizational mechanisms developed in the next section were not selected to exhaust the organizational tier of ecology; they were selected as a parsimonious, high-leverage starting set against four criteria. First, each is empirically grounded in adjacent, mature research literature, so that propositions about it can be tested using validated constructs rather than invented ones. Second, each occupies a mediating position between leader behavior and the frontline decision, consistent with the work-system logic of SEIPS 2.0: it is a condition that leaders shape and that staff then carry into the crisis moment, rather than a fixed structural constraint. Third, each is leader-modifiable, an actionable lever rather than a demographic or fiscal given. Fourth, together they span the three distinct pathways through which organizational conditions reach a discretionary decision: the relational (whether staff will seek help and disclose honestly), the normative (what staff believe is expected of them), and the informational (what staff know going into the moment). Other organizational descriptors plainly belong to the same ecology (staffing stability and turnover, supervisory dosage, reward and feedback contingencies, workload, and the physical environment among them), and a fuller model will incorporate them. The claim here is not that three variables are sufficient, but that these three are defensible, measurable, and consequential enough to anchor a testable account and to organize a practice agenda that leaders can act on now.

## Leadership as decision architecture: three organizational mechanisms

Three mechanisms are proposed through which leader behavior shapes staff decision-making patterns, each with empirical support and each specifying a distinct pathway to restraint outcomes. Within the Staff Decision Ecology Model, these three mechanisms are the organizational-psychology constructs that generate the organizational contingencies tier: they determine whether policy is enforced consistently, how supervisors respond to restraint and non-restraint decisions, what peer norms take hold, and whether alternative supports are perceived as available.

### Psychological safety

Edmondson (1999) defined team psychological safety as a shared belief that the team is safe for interpersonal risk-taking. In psychologically safe teams, members report errors, raise concerns, and seek help without anticipating punishment or marginalization, a construct with substantial empirical support across healthcare and organizational contexts (Frazier et al., 2016). In human services settings, psychological safety operates through two restraint-relevant pathways. The first is data integrity: restraint reduction depends on accurate incident reporting, and in low-safety organizations staff systematically underreport incidents to avoid punitive responses, concealing the patterns the organization most needs to see. The second is help-seeking before crisis narrows options to restraint. Nembhard and Edmondson (2006) demonstrated that leader inclusiveness (explicit invitations to voice concerns) is a significant antecedent of psychological safety, with effects amplified for lower-status team members. Direct support professionals occupy among the lowest rungs of the human services hierarchy. Their willingness to seek help during escalation is not a training variable; it is a relational one, built by leadership through cumulative behavioral signals.

Ma et al.’s (2023) structural equation modeling found subjective norm to be a significant independent predictor of restraint intention (*β* = 0.25, *p* < 0.05). Subjective norm (what staff believe their organizational community expects them to do) is constituted by those same accumulated signals. It predicts restraint independently of skill, knowledge, and individual attitude.

### Implementation climate

Implementation climate refers to shared employee perceptions of the extent to which innovation use is expected, supported, and rewarded within an organization (Weiner et al., 2011). Weiner (2009) proposed that organizational readiness for change comprises change commitment and change efficacy, both shaped by leader behavior, not by training content. An organization can provide high-quality PBS training while simultaneously maintaining an implementation climate in which trained practices are not modeled by supervisors, not recognized in feedback, and not supported by clinical resources. In that climate, staff default under pressure to familiar responses, which in many settings means restraint. Konstantinidou et al. (2023) found that the most robust predictor of PBS implementation maintenance was not training dosage but the organizational systems surrounding training: supervisory follow-up, feedback mechanisms, and leadership modeling. Deveau et al. (2020) found that leaders who actively directed organizational attention toward restrictive practice reduction achieved better outcomes than those focused primarily on compliance and audit. Björne et al. (2023) found that front-line managers reported significantly lower restraint rates than staff, evidence that organizational culture was not supporting transparent disclosure and that managers lacked accurate situational awareness of practice environments.

### Team communication structures

Dalton et al. (2023) identified team communication as one of the most significant proximal organizational predictors of restraint-related outcomes in a children’s hospital investigation. Within the decision ecology, communication structures function as pre-crisis intervention: they determine whether teams approach escalating situations with shared knowledge of who is at elevated risk, what antecedent conditions are active, and who should be called. Teams with structured processes for behavioral data review, shift handoff, and incident follow-up carry more complete situational awareness into crisis moments than teams without them. Post-restraint debriefing carries disproportionate leverage: conducted with psychological safety and a systemic orientation, debriefs determine whether incidents generate organizational learning or organizational silence. Leaders who treat debriefs as blame-allocation exercises teach staff that honesty is costly. Those who treat them as learning events build the capacity to prevent recurrence.

## A conceptual model and research agenda

**Figure 1:**
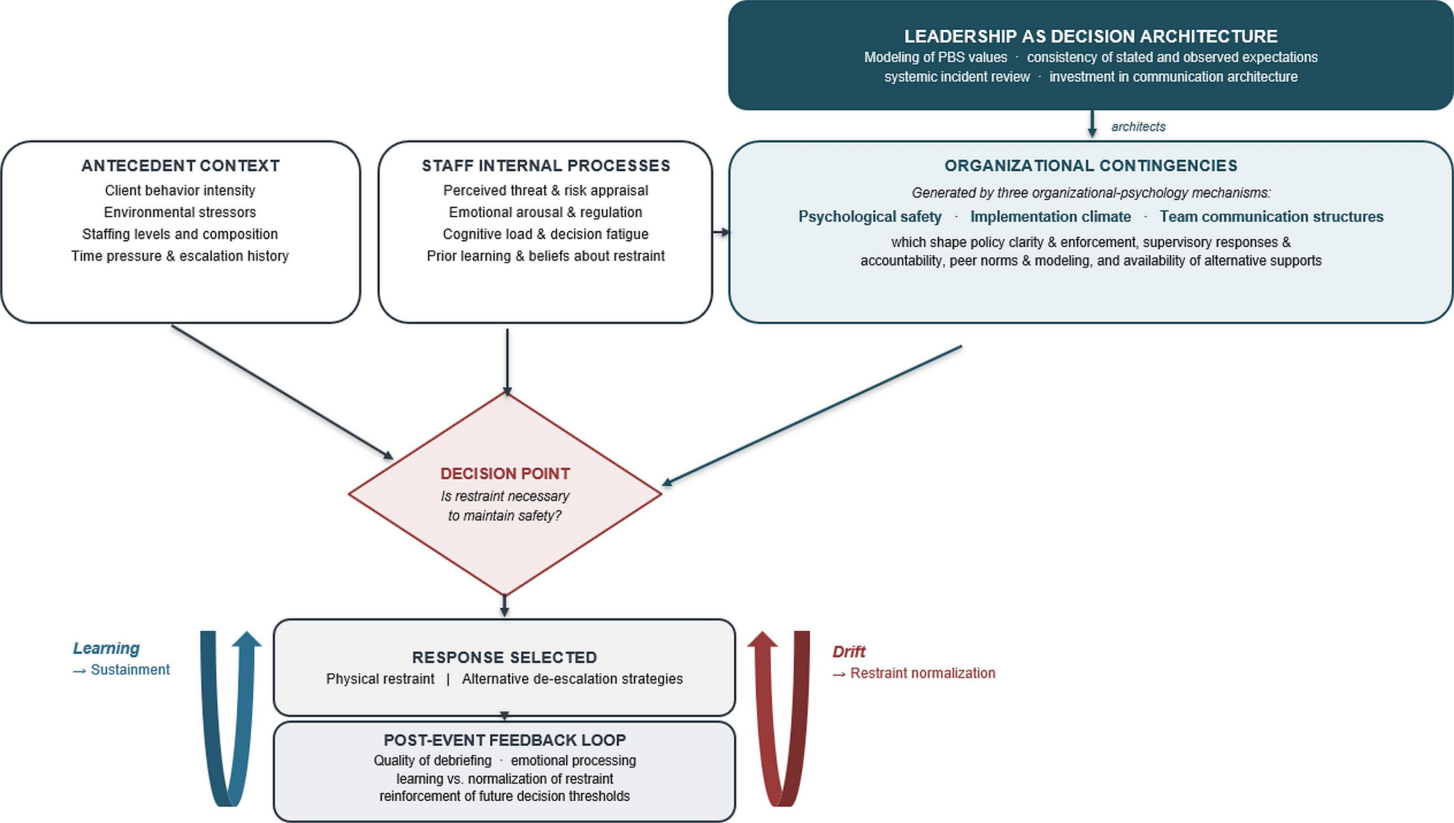
Leadership as decision architecture within the Staff Decision Ecology Model. The tiers (antecedent context, staff internal processes, and organizational contingencies), the decision point, the response selected, and the post-event feedback loop follow the Staff Decision Ecology Model, in which restraint is a staff-mediated decision that either sustains restraint-free practice (learning) or drifts toward restraint normalization. The present framework adds leadership as the architect of the organizational tier and identifies the three organizational-psychology mechanisms (psychological safety, implementation climate, and team communication structures) through which leadership generates the organizational contingencies that bear on the decision point. Staff decision-making remains the proximal mechanism; leadership operates by shaping the conditions under which that decision is made.

The three mechanisms (psychological safety, implementation climate, and team communication structures) are proposed as mediating variables between leadership behavior and restraint outcomes within the Staff Decision Ecology Model (Figure 1). Leader behaviors, specifically modeling of PBS values, consistency between stated and observed expectations, systemic orientation in incident review, and investment in communication architecture, shape the organizational tier of the decision ecology. They do so by building or eroding psychological safety, implementation climate, and communication quality. These conditions in turn shape what individual staff carry into crisis moments: the options they perceive as available, the behaviors they perceive as normatively expected, and the risks they perceive in departing from trained practice. The cumulative effect on behavioral decision-making across staff and time is what organizations measure as their restraint rate.

The model generates testable propositions. Does team psychological safety moderate the relationship between training quality and PBS fidelity at the unit level? Does implementation climate mediate the relationship between identifiable leader behaviors and organizational restraint rates? Do structured pre-crisis communication processes predict restraint rates independent of training dosage? Does the perceptual gap between manager-reported and staff-reported restraint rates, documented by Björne et al. (2023), moderate the effect of organizational interventions? These hypotheses are amenable to multilevel modeling using Edmondson’s Team Psychological Safety Scale, Weiner’s Organizational Readiness for Implementing Change (ORIC) measure, and SEIPS 2.0 adaptations for human services. The meta-analytic work of Hoch et al. (2018) on leadership styles and organizational climate provides a further empirical foundation for identifying which leader behaviors most efficiently build the organizational conditions the model specifies.

Specifying how these propositions could be tested clarifies what kind of evidence the framework demands. Because the model predicts both a clinical outcome (the restraint rate) and an implementation outcome (whether PBS practices are actually used as intended), it is well suited to effectiveness-implementation hybrid designs (Curran et al., 2012), which evaluate the two simultaneously rather than sequentially. A type 2 or type 3 hybrid would allow a restraint reduction initiative to be tested for its effect on restraint incidence while the organizational change strategy that delivers it is itself studied for adoption, fidelity, and sustainability. At the level of design, the unit rather than the individual is the appropriate target of assignment: a stepped-wedge cluster-randomized rollout, in which units or sites cross from control to intervention at randomized intervals, would permit a direct test of the framework’s central sequencing claim, that building psychological safety and implementation climate before delivering training produces larger and more durable restraint reductions than a training-first sequence. Restraint counts would be modeled as over-dispersed event data (for example, incidence rate ratios from mixed-effects Poisson or negative-binomial models) with staff nested in units and units nested in organizations, and the proposed mediators measured with established instruments (Edmondson’s Team Psychological Safety Scale, Weiner’s ORIC measure, and SEIPS 2.0 adaptations for human services), alongside Proctor et al.’s (2011) implementation outcomes (fidelity, penetration, and sustainability) as intermediate endpoints. Framing the work this way also disciplines reporting: the organizational determinants the initiative targets can be specified using the Consolidated Framework for Implementation Research (Damschroder et al., 2009), and the discrete strategies used to change them (facilitation, audit and feedback, leadership engagement, and the like) can be named and reported using the Expert Recommendations for Implementing Change compilation (Powell et al., 2015), so that a successful program can be replicated rather than admired. This is the level of specificity at which a decision ecology account becomes a research program rather than a reframing.

## A practice agenda

Leaders who wish to build psychological safety must do so through identifiable, repeatable behaviors. Nembhard and Edmondson (2006) identify leader inclusiveness (explicitly inviting frontline input and receiving concerns with curiosity rather than defensiveness) as the most empirically supported antecedent. Organizations should assess baseline psychological safety at the team level using validated instruments before implementing restraint reduction initiatives; low-safety settings require a different intervention sequence, as training before safety-building accelerates underreporting rather than improving it. Post-restraint debrief protocols should be redesigned to open with systemic questions before individual ones and to produce documented organizational action items rather than individual counseling records.

Implementation climate is built through the accumulation of visible leader behaviors across four categories. Presence: leaders must be regularly observable in practice environments, not primarily in offices and meetings. Feedback: PBS fidelity data must be shared transparently and used for learning rather than performance management. Resource allocation: clinical support and staffing ratios must be treated as restraint reduction variables, not cost centers; Björne et al. (2023) found resource limitations identified as the primary barrier above training deficits. Modeling: leaders’ own behavior with staff must reflect the PBS values articulated in policy. McGill et al. (2018) demonstrated that combining training with explicit organizational change processes including leadership modeling produced reductions in challenging behavior and associated restrictive responses that training alone had not achieved.

Communication infrastructure requires three structural investments: regular structured behavioral data review meetings at minimum weekly; standardized shift handoff protocols including explicit behavioral risk information; and a tiered consultation access system allowing frontline staff to reach clinical support before crisis escalates. The absence of accessible consultation is among the most consistent organizational risk factors in the restraint literature (Deveau et al., 2020; Björne et al., 2023). Its presence communicates that non-restrictive practice is organizationally supported, the core signal of a strong implementation climate.

Two cross-cutting commitments make this agenda actionable rather than aspirational. The first is measurement before mobilization: organizations should establish a baseline using validated instruments (a team-level psychological safety measure and an organizational readiness measure) and should treat restraint incidence as a tracked rate rather than a periodically tabulated count, because a sequence that is right for a high-safety setting can be actively harmful in a low-safety one, where training delivered before safety-building accelerates underreporting. The second is naming the change strategy. The practice moves above are not free-floating exhortations; each corresponds to an identifiable implementation strategy in the Expert Recommendations for Implementing Change compilation (Powell et al., 2015): facilitation, audit and feedback delivered for learning, leadership engagement, and the development of consultative and communication infrastructure. Organizations that specify which strategies they are deploying, against which determinants (Damschroder et al., 2009), can evaluate and replicate their own results rather than attributing change to unspecified effort. The decision ecology does not ask leaders to do more; it asks them to do identifiable things and to measure whether the organizational conditions those things are meant to build are in fact being built.

## Discussion

Physical restraint will not be eliminated by training alone. This paper has proposed the Staff Decision Ecology Model, which accounts for restraint as an organizational behavior, and has identified leadership as the primary organizational actor capable of creating the conditions that restraint-free practice requires. Psychological safety, implementation climate, and team communication structures are the proposed mechanisms, each grounded in adjacent research literatures, each generating testable hypotheses at the intersection of leadership science, implementation science, and organizational psychology. Leadership is positioned here as the architect of the organizational tier, not as a replacement for staff decision-making: consistent with the Staff Decision Ecology Model, the staff decision remains the proximal mechanism, and leadership matters precisely because it shapes the conditions under which that decision is made.

The field’s resistance to this reframing is understandable. Organizational accountability is harder than individual accountability. The training model permits attribution of persistent restraint to individual staff who failed to apply what they were taught. The decision ecology model does not permit that attribution as cleanly. It asks leaders to examine what organizational conditions they have built (or neglected to build) around the staff members who used restraint. That examination requires organizational self-scrutiny that many settings have not been structured to support.

Although restraint is the focus here, the decision ecology is not specific to it. The framework is, at its core, an account of how organizations shape high-stakes, discretionary decisions made by frontline staff under time pressure and uncertainty, and the same architecture should apply wherever those conditions hold. Seclusion, the use of as-needed psychotropic medication, the management of elopement, and the response to staff injury or to suspected abuse and neglect are all decisions of this kind: each is made in the moment by the lowest-status members of the organization, each is shaped by what those staff believe is available, expected, and safe, and each is currently theorized (as restraint long was) primarily as a matter of individual skill or compliance. Porting the framework to a new problem requires three adjustments rather than a redesign. The individual-level decision variables must be re-specified for the target behavior; the relevant environmental constraints must be re-identified; and the outcome metric must be re-anchored. The three organizational mechanisms are hypothesized to transfer, because they operate on the relational, normative, and informational substrate common to discretionary frontline decisions, but their relative weight is an empirical question that will differ by behavior and should be estimated, not assumed. The framework’s boundary conditions follow from the same logic: it has the most to offer where decisions carry genuine discretion and are made within a frontline-leadership hierarchy, and the least where decisions are fully protocolized or removed from frontline judgment.

But the cost of avoiding it is borne by the people who are restrained. Individuals with IDD who experience physical restraint face documented risks of physical injury, psychological harm, and trauma regardless of staff intent (Nunno et al., 2022; McDonnell et al., 2023). Every organizational year in which restraint rates remain elevated despite stated commitment to reduction represents a failure to act on what the evidence has established: the problem is organizational, the mechanisms are identifiable, and the solutions are buildable. The organizations that support individuals with IDD are making leadership choices right now, about how they respond to incidents, what implementation climate their behavior communicates, and what communication structures they invest in. Those choices, accumulated across leaders and time, are what the data will eventually reveal as either enabling or obstructing the restraint elimination that every mission statement in this field already claims to pursue.

## Data Availability

The original contributions presented in the study are included in the article/supplementary material, further inquiries can be directed to the corresponding author.

## References

[ref1] Björne P. McGill P. Deveau R. Hofvander B. (2023). Organisational impact on the use of restrictive measures: the perspective of Swedish front-line managers. J. Appl. Res. Intellect. Disabil. 36, 1025–1033. doi: 10.1111/jar.13112, 37151147

[ref2] Curran G. M. Bauer M. Mittman B. Pyne J. M. Stetler C. (2012). Effectiveness-implementation hybrid designs: combining elements of clinical effectiveness and implementation research to enhance public health impact. Med. Care 50, 217–226. doi: 10.1097/MLR.0b013e3182408812, 22310560 PMC3731143

[ref3] Dalton E. M. Worsley D. Krass P. Kovacs B. Raymond K. Feudtner C. . (2023). Factors influencing agitation, de-escalation, and physical restraint at a children’s hospital. J. Hosp. Med. 18, 693–702. doi: 10.1002/jhm.13159, 37401165 PMC10529788

[ref4] Damschroder L. J. Aron D. C. Keith R. E. Kirsh S. R. Alexander J. A. Lowery J. C. (2009). Fostering implementation of health services research findings into practice: a consolidated framework for advancing implementation science. Implement. Sci. 4:50. doi: 10.1186/1748-5908-4-50, 19664226 PMC2736161

[ref5] Deveau R. Gore N. McGill P. (2020). Senior manager decision-making and interactions with frontline staff in intellectual disability organisations: a Delphi study. Health Soc. Care Community 28, 81–90. doi: 10.1111/hsc.12842, 31482622

[ref6] Edmondson A. C. (1999). Psychological safety and learning behavior in work teams. Adm. Sci. Q. 44, 350–383. doi: 10.2307/2666999

[ref7] Frazier M. L. Fainshmidt S. Klinger R. L. Pezeshkan A. Vracheva V. (2016). Psychological safety: a meta-analytic review and extension. Pers. Psychol. 70, 113–165. doi: 10.1111/peps.12183

[ref8] Haines-Delmont A. Goodall K. Duxbury J. Tsang A. (2022). An evaluation of the implementation of a ‘no force first’ informed organisational guide to reduce physical restraint in mental health and learning disability inpatient settings in the UK. Front. Psych. 13:749615. doi: 10.3389/fpsyt.2022.749615, 35185645 PMC8851567

[ref9] Hayward B. Gale E. McIntyre L. (2021). Introducing positive behaviour support (PBS) into disability services for successful adoption: a synthesised systematic review. Br. J. Learn. Disabil. 50, 15–29. doi: 10.1111/bld.12363

[ref10] Hoch J. E. Bommer W. H. Dulebohn J. H. Wu D. (2018). Do ethical, authentic, and servant leadership explain variance above and beyond transformational leadership? A meta-analysis. J. Manag. 44, 501–529. doi: 10.1177/0149206316665461

[ref11] Holden R. J. Carayon P. Gurses A. P. Hoonakker P. Hundt A. S. Ozok A. A. . (2013). SEIPS 2.0: a human factors framework for studying and improving the work of healthcare professionals and patients. Ergonomics 56, 1669–1686. doi: 10.1080/00140139.2013.838643, 24088063 PMC3835697

[ref13] Konstantinidou I. Dillenburger K. Ramey D. (2023). Positive behaviour support: a systematic literature review of the effect of staff training and organisational behaviour management. Int. J. Dev. Disabil. 69, 29–44. doi: 10.1080/20473869.2022.2123199, 36743321 PMC9897795

[ref14] Ma Y. Cui N. Li X. Li J. Zhang Y. Liu Y. . (2023). Predicting critical care nurses’ intention to use physical restraints in intubated patients: a structural equation model. J. Nurs. Manag. 2023:3286312. doi: 10.1155/2023/3286312, 40225669 PMC11919005

[ref15] Mahon D. Walsh E. Holloway J. Lydon H. (2021). A systematic review of training methods to increase staff’s knowledge and implementation of positive behaviour support in residential and day settings for individuals with intellectual and developmental disabilities. J. Intellect. Disabil. 26, 732–757. doi: 10.1177/17446295211022124, 34219540 PMC9442775

[ref16] McDonnell A. A. O’Shea M. C. Bews-Pugh S. J. (2023). Staff training in physical interventions: a literature review. Front. Psych. 14:1129039. doi: 10.3389/fpsyt.2023.1129039 PMC10411725 37564241

[ref17] McGill P. Vanono L. Hewlett C. Smyth E. Joyce C. O’Brien Y. . (2018). Reducing challenging behaviour of adults with intellectual disabilities in supported accommodation: A cluster randomized controlled trial of setting-wide positive behaviour support. Res. Dev. Disabil. 81, 143–154. doi: 10.1016/j.ridd.2018.04.020 29752027

[ref18] Nembhard I. M. Edmondson A. C. (2006). Making it safe: the effects of leader inclusiveness and professional status on psychological safety and improvement efforts in health care teams. J. Organ. Behav. 27, 941–966. doi: 10.1002/job.413

[ref12] Ntinas K. M. (2024). Staff burnout in intellectual disability services and resistance to change: implications for leadership support. J. Intellect. Disabil.. (Advance online publication). doi: 10.1177/17446295241280697 39208442

[ref19] Nunno M. A. McCabe L. A. Izzo C. V. Smith E. G. Sellers D. E. Holden M. J. (2022). A 26-year study of restraint fatalities among children and adolescents in the United States: a failure of organizational structures and processes. Child Youth Care Forum 51, 661–680. doi: 10.1007/s10566-021-09646-w

[ref20] Powell B. J. Waltz T. J. Chinman M. J. Damschroder L. J. Smith J. L. Matthieu M. M. . (2015). A refined compilation of implementation strategies: results from the expert recommendations for implementing change (ERIC) project. Implement. Sci. 10:21. doi: 10.1186/s13012-015-0209-1, 25889199 PMC4328074

[ref21] Proctor E. Silmere H. Raghavan R. Hovmand P. Aarons G. Bunger A. . (2011). Outcomes for implementation research: conceptual distinctions, measurement challenges, and research agenda. Adm. Policy Ment. Health Ment. Health Serv. Res. 38, 65–76. doi: 10.1007/s10488-010-0319-7, 20957426 PMC3068522

[ref22] Weiner B. J. (2009). A theory of organizational readiness for change. Implement. Sci. 4:67. doi: 10.1186/1748-5908-4-67, 19840381 PMC2770024

[ref23] Weiner B. J. Belden C. M. Bergmire D. M. Johnston M. (2011). The meaning and measurement of implementation climate. Implement. Sci. 6:78. doi: 10.1186/1748-5908-6-78, 21781328 PMC3224582

